# The alternative value of thyroid stimulating hormone instead of thyroglobulin in differentiation of follicular thyroid neoplasm in Hashimoto’s thyroiditis

**DOI:** 10.3389/fonc.2024.1395202

**Published:** 2024-09-09

**Authors:** Jinyue Liu, Jie Kuang, Hanxing Sun, Lingxie Chen, Qinyu Li, Ling Zhan, Ri Hong, Rui Li, Jiqi Yan, Weihua Qiu, Zhuoran Liu

**Affiliations:** ^1^ Department of General Surgery, Ruijin Hospital, Shanghai Jiao Tong University School of Medicine, Shanghai, China; ^2^ Department of Pathology, Ruijin Hospital, Shanghai Jiao Tong University School of Medicine, Shanghai, China

**Keywords:** follicular thyroid carcinoma, follicular thyroid adenoma, Hashimoto’s thyroiditis, thyroglobulin, thyroid stimulating hormone

## Abstract

**Purposes:**

To provide novel aspects for the preoperative diagnosis and appropriate differentiation strategies for follicular thyroid carcinoma (FTC) and follicular thyroid adenoma (FTA).

**Methods:**

Among 25,765 cases, a total of 326 patients with follicular thyroid neoplasms between 2013 and 2019 were enrolled. Patient demographics, perioperative parameters, surgical profiles and oncologic outcomes were collected and analyzed.

**Results:**

There were no significant differences in preoperative ultrasound findings between FTA and FTC patients. The true positive rate (sensitivity) and true negative rate (specificity) of fine needle aspiration (FNA) for FTA patients were 0.6956 and 0.5000, respectively, and those for FTC patients were 0.0714 and 0.9348, respectively. Patients with FTC presented significantly higher serum thyroglobulin (TG) levels than patients with FTA. Preoperative TG level was positively related to tumor invasiveness and recurrence or distant metastases in FTC patients. There were 55 patients with Hashimoto’s thyroiditis (HT), accounting for 16.87% of enrolled patients. HT patients had significantly lower serum TG concentrations than antibody-negative patients. Among HT patients, no significant differences were observed in TG levels between the FTA and FTC groups. Instead, FTA patients had significantly higher serum thyroid stimulating hormone (TSH) levels and lower serum T3 (Triiodothyronine) levels compared to FTC patients. Serum TSH level >1.736U/L was associated with benign follicular neoplasms in HT patients according to the receiver operating characteristic (ROC) curve.

**Conclusion:**

Distinguishing FTC from FTA remains a challenge for ultrasonography and FNA. Serum TG should be measured as a risk factor of FTC. However, in HT patients, serum TSH levels can serve as a more reliable indicator for differentiating FTC from FTA preoperatively.

## 1 Introduction

The incidence of thyroid cancer has increased dramatically over the past few decades in the world, with an estimated 43,720 new cases a year in the United States (1). As the second most common type of differentiated thyroid cancer (DTC), the incidence of follicular thyroid carcinoma (FTC) is much lower than that of papillary thyroid carcinoma (PTC) (2). Compared to PTC, patients with FTC have a two-fold and ten-fold higher risk of developing lung and bone metastases, respectively, due to its propensity for vascular invasion and hematogenous dissemination (3). Therefore, the outcome of FTC is less favorable than that of PTC (4–6). FTC is defined as a malignant epithelial tumor showing follicular cell differentiation, without the nuclear features of PTC (7). Preoperative examinations, such as ultrasonography and fine needle aspiration (FNA), are usually not sufficient to distinguish FTC from its benign counterpart, follicular thyroid adenoma (FTA) (8–10). The histological differences between FTC and FTA are capsular and vascular invasion (10). Thus, it remains challenging for clinicians to determine appropriate extent of therapy and to avoid unnecessary surgery in patients diagnosed with follicular thyroid neoplasms. Several factors have been reported to be associated with the malignancy of a follicular neoplasm, such as large tumor size, but the diagnosis of FTC still mostly relies on postoperative pathology (11). More research may be required to better evaluate the risk of malignancy in patients with follicular thyroid neoplasm.

As one of the most common thyroid diseases, Hashimoto’s thyroiditis (HT) was first described by a Japanese physician, Haraku Hashimoto in 1912. It is the most frequent autoimmune thyroid disease characterized by enlarged thyroid volume, parenchymal lymphocytic infiltration, and thyroid-specific autoantibodies (12). Currently, the incidence of HT is 0.3-1.5 cases per 1000 people, and it has been constantly increasing in recent years (13–17). Women are 4-10 times more likely to have HT than men (17). Circulating antibodies to thyroperoxidase and thyroglobulin are two serological markers of HT, which are widely used in clinical diagnosis (18). Most HT may cause endocrine disorder and ultimately develop to hypothyroidism, although at presentation patients can be euthyroid or hyperthyroid (15, 19). Although several reports have confirmed that HT could be associated with a higher risk of PTC, PTC combined with HT have less metastasis and better outcomes of PTC than patients without HT (20, 21). As to FTC, less relationship between HT and FTC has been established yet.

In this study, the clinical features of patients with follicular thyroid neoplasms were analyzed to portrait the differences between FTA and FTC. Then, the influence of HT on FTC diagnosis and differentiation was further investigated. Our findings are expected to explore new aspects for the preoperative diagnosis and appropriate differentiation strategies in patients with follicular thyroid neoplasms.

## 2 Materials and methods

### 2.1 Patient recruitment

There were 25,765 patients with thyroid tumors received initial surgery at the Department of General Surgery, Ruijin Hospital Shanghai Jiao Tong University School of Medicine from 2013 to 2019. Our series included 19,069 malignant and 6,696 benign tumors. Patients meeting any of the following criteria were excluded from our study: 1) non-follicular thyroid neoplasms pathological type; 2) having received thyroid-related surgery previously; 3) history of neck radiation. 4) performed LT4 (Levothyroxine) therapy before. According to these criteria, a total of 326 patients were enrolled in current study. Patient demographics, perioperative parameters, surgical profiles and oncologic outcomes were collected and analyzed. The informed consent form was signed by every patient to indicate that they agreed to undergo the operation and to the use of the perioperative data. The research consent was approved by the Ethics Committee and the Institutional Review Board of Shanghai Ruijin Hospital (Approval No. RHE-D-2021-024).

### 2.2 Clinicopathological features and follow-up visits

Patients demographics including age, gender, primary tumor size, preoperative serum concentrations of thyroid hormones, TG, TGAb (Thyroid Stimulating Hormone), and TPOAb (Thyroid Peroxidase Antibodies), were retrospectively collected. HT was diagnosed when TGAb>4.11 IU/mL or TPOAb>5.61 IU/mL. Preoperative neck ultrasonography was performed in all patients, while some patients received FNA due to suspicious malignant nodules. All enrolled patients were diagnosed as FTA or FTC by postoperative pathological examination. The follow-up visits were initiated at the fourth week postoperatively. Biochemical investigations were estimated on the first postoperative visit. All patients with complete follow-up accepted ultrasonographic examination every six months postoperatively for recurrence estimation. Recurrence and distant metastases were focused on in FTC patients.

### 2.3 Statistical analysis

Data were analyzed using R software (ver. 3.5) and SPSS 23.0 statistical packages. Continuous variables were described using mean and standard deviation and were compared using Student’s *t* test or one-way ANOVA test followed by Least Significant Difference (LSD) test. Categorical variables were described as number and percentage, and the percentage of categorical variables among groups were compared using Chi-square test. P-values < 0.05 was accepted as statistically significant. Receiver-operating characteristic (ROC) curve analysis was performed using pROC packages of R software (22, 23).

## 3 Results

### 3.1 Thyroid ultrasonography of FTA and FTC

**Figure 1:**
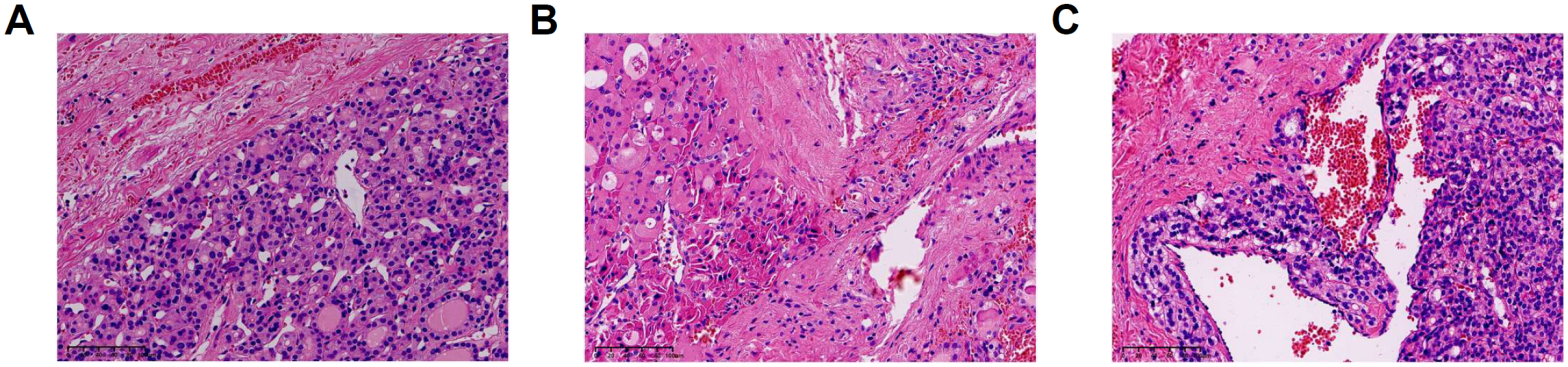
Pathological features of FTA (Follicular Thyroid Adenoma) and FTC (Follicular Thyroid Carcinoma) (hematoxylin and eosin staining). **(A)** FTA **(B)** FTC with capsular invasion. **(C)** FTC with vascular invasion.

Totally 201 patients with FTA and 125 patients with FTC were included in this study. The diagnoses were confirmed by pathological examination (**Figure 1**). All patients enrolled were received neck ultrasonography before operation and were classified according to the Thyroid Imaging and Reporting Data System (TIRADS) (24). As shown in **Table 1**, 218 in 326 were reported as “TIRADS 4A”, accounting for 66.9%. Among patients with TIRADS 4A, 118 patients were considered as “possible follicular tumor”. There were 74 cases (62.7%) in FTA group and 44 cases (37.3%) in FTC group. According to *Chi*-square test, there was no significant difference in preoperative ultrasound TIRADS classification to differentiate FTA from FTC (p=0.461). In addition, no significant variance could be identified between FTA and FTC groups in terms of ultrasonic characters, such as shape, margin, echogenicity, echogenic foci, and the degree of blood supply, and the p values were 0.591, 0.465, 0.150, 0.212, and 0.359, respectively.

**Table 1:** Comparison of thyroid ultrasonography in patients with FTA and FTC.

	FTA(n=201)	FTC(n=125)	P-value
US-TIRADS, n (%)			0.461
3	51(56.7)	39(43.3)	
4A	65(65.7)	34(34.3)	
4A, possible follicular tumor	74(62.7)	44(37.3)	
4A, possible source of parathyroidea	1(100)	0(0)	
4B	6(66.7)	3(33.3)	
4C	0(0)	2(100)	
5/possible cancer	4(57.1)	3(42.9)	
shape, n (%)			0.591
oval	196(61.4)	123(38.6)	
irregular	5(71.4)	2(28.6)	
margin, n (%)			0.465
smooth	24(53.3)	21(46.7)	
basically smooth	172(63.0)	101(37.0)	
irregular	5(62.3)	3(37.5)	
homogeneous echo, n (%)			0.070
yes	20(48.8)	21(51.2)	
no	181(63.5)	104(36.5)	
echogenicity, n (%)			0.150
very hypoechoic	0(0)	1(100)	
hypoechoic	97(58.1)	70(41.9)	
isoechoic	36(75)	12(25)	
mixed-echoic	67(61.5)	42(38.5)	
hyperechoic	1(100)	0(0)	
calcification echogenic foci, n (%)			0.212
no	145(63.9)	82(36.1)	
yes	56(56.6)	43(43.4)	
Blood supply, n (%)			
poor	54(60.7)	35(39.3)	0.359
medium	66(67.3)	32(32.7)	
rich	81(58.3)	58(41.7)	

FTA, follicular thyroid adenoma.; FTC, follicular thyroid carcinoma.

### 3.2 FNA of FTA and FTC

There were 74 patients received FNA before operation, including 46 cases in FTA group and 28 cases in FTC group. Among 46 FTA patients, 32 patients were truly reported as “FTA”, and 9 patients were considered as “nodular goiter”. Three patients were diagnosed as “FTC”, and 2 patients were reported as “PTC”. The sensitivity and specificity are 0.6956 and 0.5000, respectively for FTA patients. Whereas in FTC group, only 2 patients were successfully diagnosed. One was reported as “FTC”, and the other as “oncocytic carcinoma”. Among the rest patients with FTC, 14 patients were faultily diagnosed as “FTA”. Eight patients were considered to be “PTC” and 4 patients were reported as “nodular goiter”. The sensitivity and specificity are 0.0714 and 0.9348, respectively for FTC patients. The comparison of FNA in patients with FTA and FTC was summarized in **Table 2**.

**Table 2:** Comparison of FNA in patients with FTA and FTC.

FNAC, n=74, n (%)	FTA(n=46)	FTC(n=28)
nodular goiter	9(69.2)	4(30.8)
FTA	32(69.6)	14(30.4)
oncocytic carcinoma	0(0)	1(100)
FTC	3(75)	1(25)
PTC	2(20)	8(80)

FNA, fine needle aspiration; FTA, follicular thyroid adenoma; FTC, follicular thyroid carcinoma; PTC, papillary thyroid carcinoma.

### 3.3 Clinicopathological features of FTA and FTC

**Figure 2:**
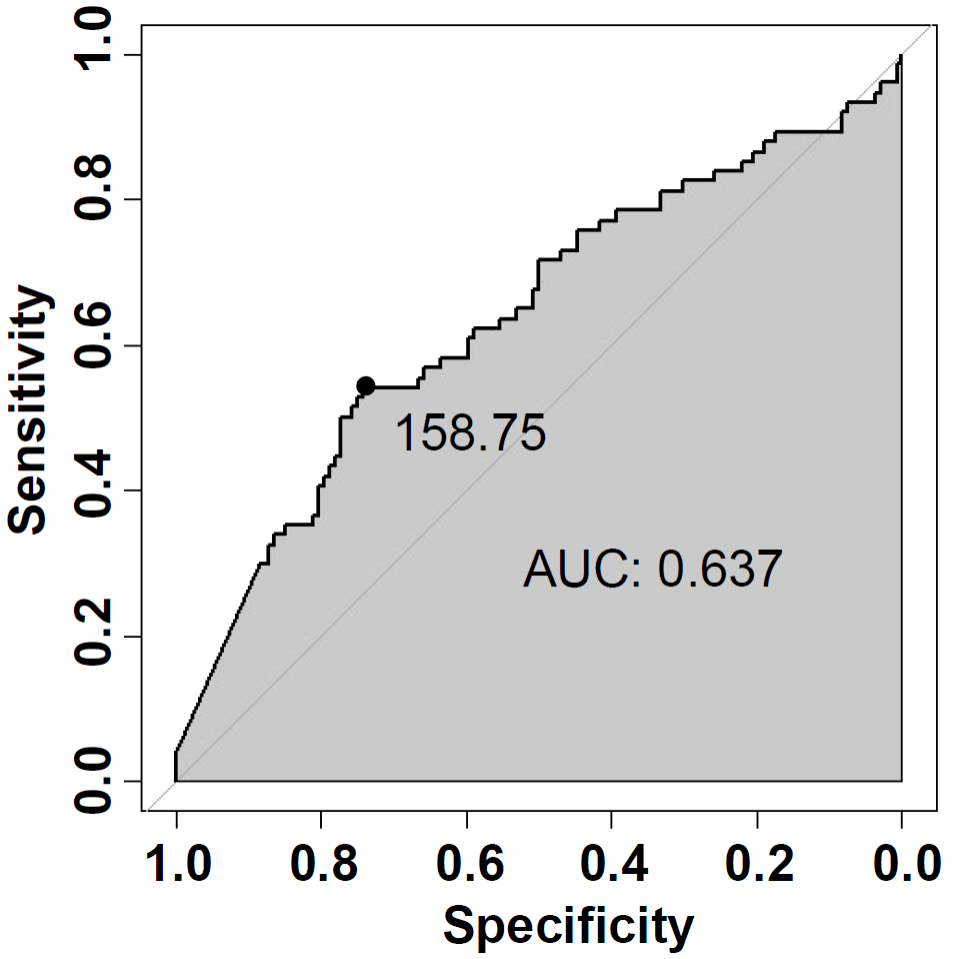
Receiver-operating characteristic (ROC) curve analysis of serum TG (Thyroglobulin) level for predicting FTC.

The basic clinicopathological features of patients with FTA or FTC were shown and compared in **Table 3**. Patients with FTC presented significantly higher serum TG levels than patients with FTA (257.9 ± 28.9 ng/mL *vs*. 138.4 ± 14.5 ng/mL, *p*=0.0011). ROC analysis was performed to evaluate the predictive accuracy of serum TG level for FTC (**Figure 2**). Serum TG level >158.75 ng/mL was associated with FTC (sensitivity 0.541, specificity 0.742), and the AUC was 0.637 (95% CI: 0.555-0.719). For other characteristics, such as gender, age, BMI, tumor size, rest thyroid-related hormone levels, and serum antibody levels, no significant differences were observed. The significant difference indicated that higher TG levels are related to the occurrence of malignant tumors.

**Table 3:** Comparison of clinicopathological features between patients with FTA and FTC.

	FTA(n=201)	FTC(n=125)	P-value
Gender, n (%)			0.55
Men	69(59.5)	47(40.5)	
Women	132(62.9)	78(37.1)	
Age, Mean ± SD (n=326)	45.8 ± 1.0	46.1 ± 1.4	0.82
Category, n (%)			0.32
≤55	144(63.4)	83(36.6)	
>55	57(57.6)	42(42.4)	
BMI (n=143)	23.1 ± 2.9	23.2 ± 3.1	0.81
Tumor size (cm, n=326)	2.81 ± 0.11	3.19 ± 0.16	0.059
VitaminD3 (nmol/L, n=180)	45.7 ± 1.7	47.5 ± 2.2	0.60
PTH (pg/ml, n=181)	57.5 ± 2.5	55.9 ± 5.5	0.23
FT3 (pmol/L, n=252)	4.36 ± 0.04	4.44 ± 0.1	0.48
FT4 (pmol/L, n=252)	13.3 ± 0.1	13 ± 0.2	0.073
TG (ng/mL, n=206)	138.4 ± 14.5	257.9 ± 28.9	0.0011^**^
TSH (μIU/mL, n=252)	2.88 ± 0.7	1.9 ± 0.38	0.052
T3 (nmol/L, n=202)	1.65 ± 0.02	1.68 ± 0.03	0.88
T4 (nmol/L, n=203)	85.6 ± 1.5	80.6 ± 2.1	0.052
TPOAb (IU/mL, n=235)	30.6 ± 10.9	28.3 ± 13.4	0.75
Category, n (%)			0.49
Elevated	19(55.9)	15(44.1)	
Normal	125(62.2)	76(37.8)	
TGAb (IU/mL, n=236)	10.14 ± 3.37	9.46 ± 4.38	0.18
Category, n (%)			0.15
Elevated	28(71.8)	11(28.2)	
Normal	117(59.4)	80(40.6)	

Error estimate represents the standard error of the mean.

^**^p<0.01 was considered statistically significant.

FTA, follicular thyroid adenoma; FTC, follicular thyroid carcinoma; BMI, body mass index; PTH, parathyroid hormone; FT3, Free Triiodothyronine; FT4, Free Thyroxine; TG, thyroglobulin; TSH, thyroid stimulating hormone; T3, Triiodothyronine; T4, Thyroxine; TPOAb, thyroid peroxidase antibody; TGAb, thyroglobulin antibody.

### 3.4 Comparison of FTC patients between pathological classification

**Figure 3:**
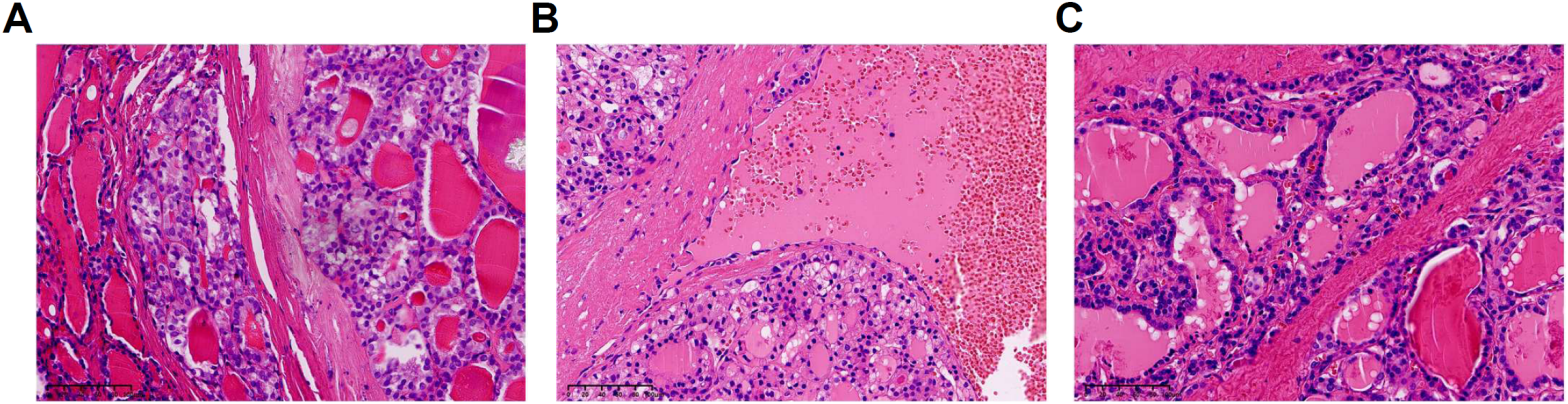
Pathological classification of FTC (Follicular Thyroid Carcinoma) (hematoxylin and eosin staining). **(A)** Minimally invasion. **(B)** Angioinvasion. **(C)** Widely invasion.

The 125 FTC patients were pathologically divided into 3 groups: minimally invasion, angioinvasion, and widely invasion group according to 2017 WHO classification of thyroid tumors (**Figure 3**). The clinical features of FTC patients among different pathological classification were shown in **Table 4**. Among the 3 groups, T4 (Thyroxine), FT4 (Free Thyroxine), TSH, and TG levels showed significant differences. Additionally, multiple comparisons were conducted using the LSD test. The results indicated that, T4 levels were significantly lower in widely invasion group than that in minimally invasion group (*p*=0.0003) and angioinvasion group (*p*=0.0016). FT4 levels of the widely invasion group were significantly lower than that in the minimally invasion group (*p=*0.005). Meanwhile, TSH levels were significantly higher in widely invasion group than that in minimally invasion group (*p=*0.0035) and angioinvasion group(*p=*0.0075). Compared to the minimally invasive group, the preoperative serum TG is significantly higher in the widely invasion group (*p=*0.0035) and the angioinvasion group (*p=*0.0075). Thus, tumor invasion was positively correlated with both TG and TSH levels in FTC patients.

**Table 4:** Comparison of FTC patients among pathological classifications.

	minimally invasion (n=92)	angioinvasion (n=22)	widely invasion (n=11)	P-value
Gender, n (%)				0.312
Men	31(66.6)	11(23.4)	5(10.6)	
Women	61(78.2)	11(14.1)	6(7.7)	
Age, Mean ± SD (n=125)	45.75 ± 14.80	48.04 ± 17.10	45.91 ± 18.99	0.824
Category, n (%)				0.442
≤55	64(77.1)	13(15.7)	6(7.2)	
>55	28(66.7)	9(21.4)	5(11.9)	
Tumor size (cm, n=125)	2.85 ± 1.51	4.31 ± 2.31	3.80 ± 1.59	0.001^**^
VitaminD3 (nmol/L, n=70)	48.41 ± 18.94	45.72 ± 17.15	43.25 ± 17.74	0.744
PTH (pg/ml, n=70)	59.24 ± 52.45	45.91 ± 21.69	46.96 ± 12.26	0.600
FT3 (pmol/L, n=96)	4.42 ± 0.56	4.47 ± 0.59	4.50 ± 1.92	0.928
FT4 (pmol/L, n=96)	13.28 ± 1.60	12.67 ± 1.84	11.55 ± 2.29	0.014^*^,b^^
TG (ng/mL, n=74)	191.45 ± 192.67	371.84 ± 312.36	457.12 ± 279.82	0.001^**^,a,b^^
TSH (μIU/mL, n=96)	1.55 ± 0.92	1.48 ± 1.18	5.37 ± 11.75	0.012^*^,b,c^^
T3 (nmol/L, n=80)	1.65 ± 0.19	1.70 ± 0.32	1.87 ± 0.71	0.203
T4 (nmol/L, n=81)	82.96 ± 13.85	83.48 ± 21.17	58.27 ± 27.95	0.001^**^,b,c^^
TPOAb (IU/mL, n=91)	23.63 ± 88.30	60.83 ± 242.06	0.82 ± 1.21	0.452
TGAb (IU/mL, n=91)	11.91 ± 49.16	3.96 ± 7.76	2.11 ± 1.44	0.676

Error estimate represents the standard error of the mean.

*p<0.05 or **p<0.01 were considered statistically significant.

a p value<0.01 between minimally invasion group and angioinvasion group.

b p value<0.01 between minimally invasion group and widely invasion.

c p value<0.01 widely invasion group and angioinvasion group.

FTC, follicular thyroid carcinoma; PTH, parathyroid hormone; FT3, Free Triiodothyronine; FT4, Free Thyroxine; TG, thyroglobulin; TSH, thyroid stimulating hormone; T3, Triiodothyronine; T4, Thyroxine; TPOAb, thyroid peroxidase antibody; TGAb, thyroglobulin antibody.

### 3.5 Follow-up outcomes of FTC patients

Totally, 94 patients (75.2%) obtained complete follow-up data (**Table 5**), and the average follow-up time was 69.13 **±** 19.56 months. Among 94 patients, recurrence or distant metastasis occurred in 6 patients, accounting for 6.38%. One patient died from FTC bone metastases 2 years after surgery, and the disease-specific mortality rate was 1.06%. Another patient died of bladder cancer 4 years after surgery, with a 5-year overall survival rate of 97.87%.

**Table 5:** Comparison of patients with or without recurrence or distant metastasis.

	without recurrence or distant metastasis (n=88)	with recurrence or distant metastasis (n=6)	P-value
Gender, n (%)			0.260
Men	35(97.2)	1(2.8)	
Women	53(91.4)	5(8.6)	
Age, Mean ± SD (n=94)	44.44 ± 14.89	40.00 ± 13.69	0.479
Category, n (%)			0.569
≤55	64(92.8)	5(7.2)	
>55	24(96.0)	1(4.0)	
Tumor size (cm, n=94)	3.18 ± 1.76	4.17 ± 1.69	0.134
Vitamin D3 (nmol/L, n=58)	48.00 ± 17.53	41.08 ± 21.92	0.456
PTH (pg/ml,n=58)	57.62 ± 52.20	55.10 ± 17.48	0.504
FT3 (pmol/L, n=75)	4.40 ± 0.67	5.23 ± 1.92	0.811
FT4 (pmol/L, n=75)	13.10 ± 1.83	10.76 ± 1.78	0.075
TG (ng/mL, n=62)	222.47 ± 223.01	625.00 ± 250.00	0.005^**^
TSH (μIU/mL, n=75)	1.58 ± 1.08	9.51 ± 17.97	0.577
T3(nmol/L, n=65)	1.65 ± 0.22	2.08 ± 0.85	0.425
T4(nmol/L, n=65)	81.88 ± 17.25	66.86 ± 14.88	0.097
TPOAB(IU/mL, n=73)	36.84 ± 145.61	1.15 ± 1.81	0.935
TGAB(IU/mL, n=73)	11.50 ± 47.73	9.15 ± 14.67	0.460

Error estimate represents the standard error of the mean.

**p<0.01 was considered statistically significant.

PTH, parathyroid hormone; FT3, Free Triiodothyronine; FT4, Free Thyroxine; TG, thyroglobulin; TSH, thyroid stimulating hormone; T3, Triiodothyronine; T4, Thyroxine; TPOAb, thyroid peroxidase antibody; TGAb, thyroglobulin antibody.

Patients were divided into two groups according to the presence of recurrence and distant metastases or not. The preoperative TG level of patients with recurrence or distant metastasis was 625.00 **±** 250.00 ng/mL, which was significantly higher than that of patients without recurrence or distant metastasis (222.47 **±** 223.01 ng/mL, p=0.005). Other characteristics, such as gender, age, tumor size, and rest thyroid-related hormones, showed no significant differences. Altogether, higher TG levels were related with recurrence and distant metastases in the FTC.

### 3.6 Unique characteristics of FTC in HT patients

According to preoperative examination, 55 patients were found to have elevated serum TPOAb or/and TGAb levels (TGAb>4.11 IU/mL, TPOAb>5.61 IU/mL) in 326 patients, which were considered to have HT. HT patients accounted for 16.87% of enrolled patients. Patients with HT had significantly lower serum TG concentrations than antibody-negative patients (114.86 **±** 23.19 ng/mL vs. 201.01 **±** 17.17 ng/mL, p=0.005). Among HT patients, there were 35 cases in FTA group and 20 cases in FTC group (**Table 6**). The prevalence of FTC was 36.36% in HT patients, and 38.75% in patients without HT, which failed to show significant difference between them (p=0.7405). Meanwhile, no significant differences were observed in TG levels (109.4 **±** 4.8 ng/mL vs. 124.5 **±** 11 ng/mL, p= 0.66) between FTA and FTC group in HT patients. Instead, FTA patients has significantly higher serum TSH level (3.76 **±** 0.2 μIU/mL vs. 1.38 **±** 0.06 IU/mL, p= 0.013) and lower serum T3 level (1.61 **±** 0.01 nmol/L vs. 1.82 **±** 0.02nmol/L, p= 0.019) compared to FTC patients. Moreover, 4 out of 5 patients with lowered TSH levels were diagnosed with FTC, while in patients with elevated TSH levels, none was diagnosed with FTC. To conclude, in HT patients, TG levels have no significant differences between the benignity and malignancy. Moreover, contrary to results above, higher TSH levels indicate benign tumors in HT patients. Therefore, our results confirmed the unique characteristics of FTC related to HT.

**Table 6:** Clinicopathological features of FTA and FTC patients with HT.

	FTA(n=35)	FTC(n=20)	P-value
Gender, n (%)			0.48
Men	6(54.5)	5(45.5)	
Women	29(65.9)	15(34.1)	
Age (n=55)	45.9 ± 0.4	45.5 ± 0.7	0.97
Category, n (%)			0.73
≤55	26(65.0)	14(35.0)	
>55	9(60.0)	6(40.0)	
BMI (n=33)	22.7 ± 0.1	22.9 ± 0.4	0.68
Tumor size (cm, n=55)	3.27 ± 0.05	3.64 ± 0.14	0.91
VitaminD3 (nmol/L, n=41)	48.9 ± 0.7	46.2 ± 1.4	0.65
PTH (pg/ml, n=41)	51.6 ± 0.9	55.6 ± 1.5	0.37
FT3 (pmol/L, n=55)	4.3 ± 0.01	4.6 ± 0.03	0.083
FT4 (pmol/L, n=55)	12.7 ± 0.1	13.6 ± 0.1	0.083
TG (ng/mL, n=47)	109.4 ± 4.8	124.5 ± 11	0.66
TSH (μIU/mL, n=55)	3.76 ± 0.2	1.38 ± 0.06	0.013^*^
Category, n (%)			0.040^*^
Lowered	1(20.0)	4(80.0)	
Normal	30(65.2)	16(34.8)	
Elevated	4(100.0)	0(0.0)	
T3 (nmol/L, n=46)	1.61 ± 0.01	1.82 ± 0.02	0.019^*^
T4 (nmol/L, n=46)	81.7 ± 0.6	86 ± 1.1	0.48
TPOAb (IU/mL, n=55)	124.5 ± 7	127.5 ± 12.6	0.46
TGAb (IU/mL, n=55)	37.4 ± 2.2	38.5 ± 4.2	0.35

Error estimate represents the standard error of the mean.

^*^p<0.05 was considered statistically significant.

FTA, follicular thyroid adenoma; FTC, follicular thyroid carcinoma; HT, Hashimoto’s thyroiditis; BMI, body mass index; PTH, parathyroid hormone; FT3, Free Triiodothyronine; FT4, Free Thyroxine; TG, thyroglobulin; TSH, thyroid stimulating hormone; T3, Triiodothyronine; T4, Thyroxine; TPOAb, thyroid peroxidase antibody; TGAb, thyroglobulin antibody.

### 3.7 Predicting benign follicular thyroid neoplasm with TSH level in HT patients

**Figure 4:**
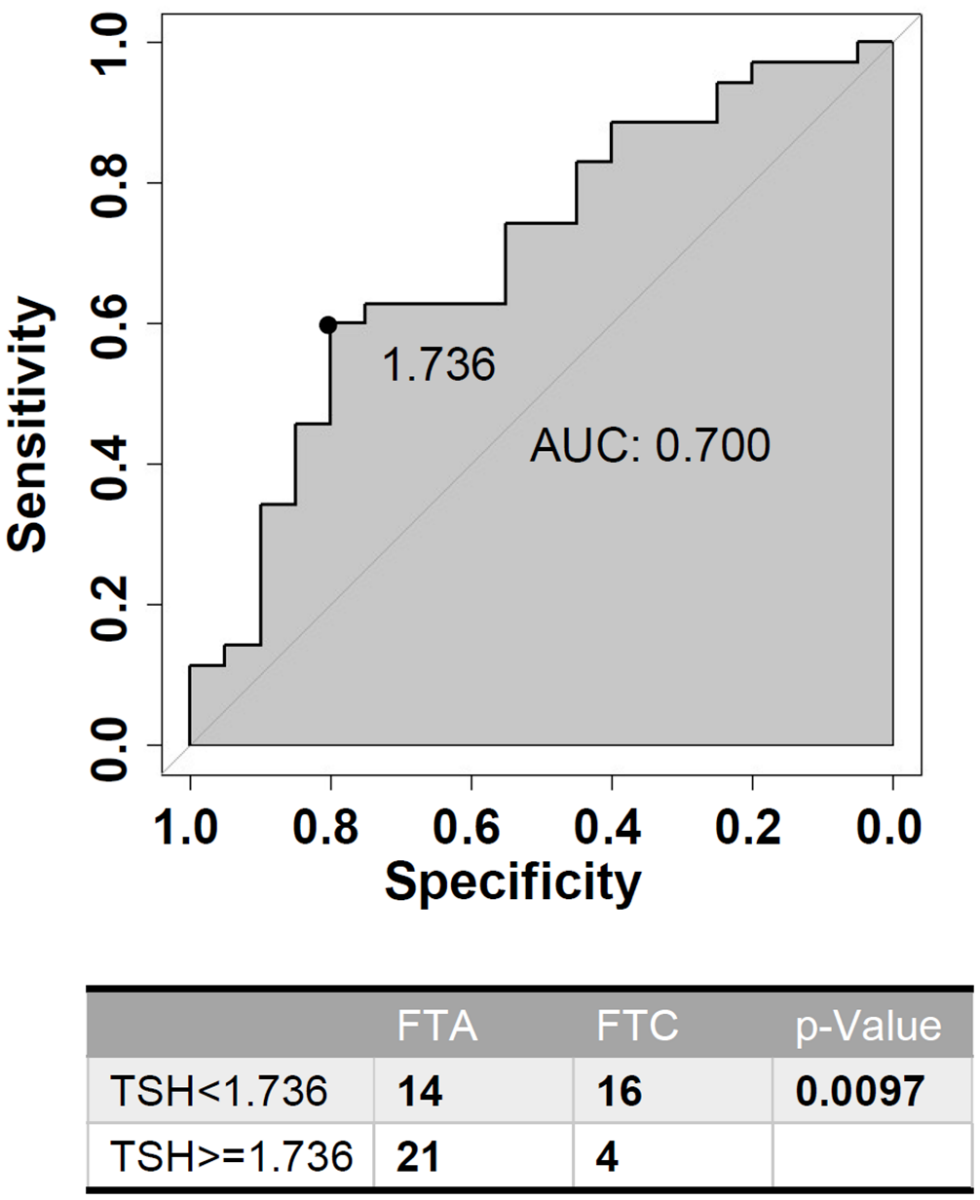
Receiver-operating characteristic (ROC) curve analysis of TSH (Thyroid Stimulating Hormone) level for predicting benign follicular neoplasms in HT (Hashimoto’s Thyroiditis) patients.

ROC analysis was performed to evaluate the predictive accuracy of TSH level for benign follicular tumor in patients with HT (**Figure 4**). Serum TSH level >1.736U/L was associated with benign follicular thyroid neoplasm (sensitivity 0.6, specificity 0.8), and the AUC was 0.7 (95% CI: 0.5535-0.8465). Evidenced by quantitative study, higher TSH levels indicate benign tumors in HT patients, which may help distinguish FTC from FTA.

## 4 Discussion

Neck ultrasonography has become the most common imaging tool for patients with thyroid disease (25). According to the TIRADS, thyroid nodules with specific ultrasound features, such as solidity, hypoechogenicity, taller-than-wide shape, irregular margins, and microcalcifications, are of high malignancy risk, which helps to identify PTC (24). However, these features are of limited value in diagnosing follicular tumors, especially when differentiating FTC from FTA. The common ultrasound presentations of these two follicular lesions are basically overlapped, including well defined, isoechoic or hypoechoic and with peripheral halo (9, 26). Although a recent paper reported that FTA were less frequently classified in the high-suspicious category compared with follicular variant of PTC and FTC by ultrasound (27), more clinical data are needed for further verification. Most studies have shown that the TIRADS classification is not helpful in differentiating FTC from FTA (28). Similarly, our research of 326 patients with follicular thyroid tumors also found that there was no significant difference in ultrasound TIRADS classification or other ultrasound features between FTA and FTC patients. These results suggested that it was difficult to distinguish FTA and FTC preoperatively solely by ultrasonography.

FNA is commonly used for preoperative judgment of thyroid nodules, which could be the most effective method for diagnosis of PTC before surgery (29). But in terms of follicular tumor diagnosis, only 2 patients were successfully diagnosed among 28 patients in FTC group, while 14 patients were misdiagnosed as “FTA”. The true positive rate (sensitivity) of FTC was only 0.0714, far below the clinical diagnostic requirements. FTCs are pathologically distinguishable from FTAs based on the capsular and vascular invasion, which can hardly be portraited in cytological specimens (10). Thus, it was challenging to distinguish FTA and FTC cytologically. Molecular testing to detect individual mutations has been used for the diagnosis and prognosis of thyroid cancer (30). Studies showed that the importance of RAS mutations detection in FTC diagnosis, with the prevalence of about 40-50% in FTC and 20-30% in FTA (31–33). However, different from the decisive role of BRAF mutation in PTC diagnosis, RAS mutations may not establish a clear cut-off boundary between FTC and FTA.

Hence, we focused on thyroid function indicators expected to clarify the diagnosis of FTC preoperatively. We found that TG levels were significantly higher in FTC patients compared to FTA, which is consistent with previous studies that the risk of DTC was positively correlated with TG levels (34–36). Furthermore, the association with TG was stronger in follicular cancer than PTC (34). Only synthetized in thyroid, TG is primarily determined by the mass of differentiated thyroid tissue present, physical damage or inflammation of the thyroid, and the magnitude of thyrotropin receptor stimulation (37). Elevated TG levels in DTC are likely due to enhanced secretion in the conversion to malignancy of the thyroid follicular cells (35). Preoperative serum TG level proved to be an effective predictor of cervical lymph node metastasis and distant metastasis in DTC (38, 39), especially in PTC (40, 41). Our data shows that high TG levels were also related with tumor invasion and poor prognosis in the FTC. Therefore, we believe that preoperative serum TG should be measured as a predictor of FTC and reference for surgery selection.

However, in our study, the lack of significant differences in TG levels between FTA and FTC patients with HT demonstrated the uniqueness of FTC related to HT. Studies suggested that the presence of TGAb in HT patients can influence the measurement of serum TG levels, which may result in reduced concentrations of TG levels (42). This is consistent with our study that the serum TG concentration of HT patients was significantly lower than that of antibody-negative patients. Therefore, TG in HT patients may be misty to reflect on the real difference between FTC and FTA. Our results were consistent with previous notion that TGAb limits the application of TG as a reference to predict malignant risk in DTC (36). On the other hand, interestingly, we confirmed that the gaps of serum TSH levels and T3 levels were much more apparent between FTC and FTA in HT patients.

As the major growth factor for thyroid follicular cells, TSH suppression is a mainstay of clinical thyroid cancer management (43). The positive correlation between TSH and tumor invasion in FTC patients was found in our study, which also confirmed the importance of TSH inhibition for patients diagnosed with thyroid cancer. However, no significant differences were observed in TSH levels between FTC and FTA in general investigation of all 326 patients. On the contrary, by more detailed stratification, HT patients with higher TSH levels were significantly associated with lower risk of FTC. The negative relationship between TSH level and thyroid cancer risk was also found in both categorical and continuous analyses and supported by a genetic predisposition research (34, 44). Low TSH levels may cause less differentiation of the thyroid epithelial, resulting in a predisposition to malignant transformation (44). In our opinion, the autoimmune response in HT patients induced thyrocyte destruction and attack benign and malignant cells indiscriminately. During this process, the damage of malignant cells may help reduce the risk of FTC. Unlike the variability of TSH among people, most HT patients go through a process that gradually develop to hypothyroidism with elevated TSH levels, which decrease the other noise to influence TSH levels. In our study, the incidence rate of FTA was higher with preoperative serum TSH >1.736U/L according to ROC curve, which suggested that TSH levels could be served as a predictor to separate benign or malignant thyroid neoplasms in HT patients. The detailed regulatory mechanism still deserves further exploration.

Our study has several limitations, including the relatively small sample size, and the underlying molecular mechanism remains unclear. Besides, to accurately differentiate FTC from FTA, a more comprehensive diagnostic approach involving serologic markers, molecular testing, and other diagnostic techniques is required. To this end, studies including larger sample sizes and multiple diagnostic techniques are still required.

## 5 Conclusion

There is still a challenge for Ultrasonography and FNA to distinguish FTC from FTA. Serum TG should be measured as a risk factor of FTC. However, in HT, serum TSH levels can be a more reliable indicator to distinguish FTC from FTA preoperatively.

## Data Availability

The raw data supporting the conclusions of this article will be made available by the authors, without undue reservation.
